# Supplementary material for: A Likelihood Approach for Real-Time Calibration of Stochastic Compartmental Epidemic Models
Source: PLoS Comput Biol. 2017 Jan 17;13(1):e1005257. doi: 10.1371/journal.pcbi.1005257 (PMC5240920; doi:10.1371/journal.pcbi.1005257)
Supplement: S1 File — (TAR.GZ) [file pcbi.1005257.s014.tar.gz › HSPH_Online-SI-Revision/PF08/n100-severe/n100-severe_PF_table8.pdf]

1	R0	{1.65278, 9.06793}	{2.29342, 20.}	{{2.27921, 20.}, {2.30491, 20.}}	{38.7615, 120.558}	inf	1287.	7852.92	{7770.83, 7929.02}	510.172	0.785439	newly	875.	2495.79	{2440., 2560.}	185.233	0.455119	Reff	0.583613	0.449016	{0.442369, 0.455151}	23.0627	ar	66 342.	98 895.6	{98 320., 99 418.}	49.0693
2	R0	{1.4321, 3.80644}	{3., 20.}	{{3., 20.}, {3., 20.}}	{109.483, 425.425}	inf	47.	6285.29	{6273.57, 6304.03}	13 273.	2.12623	newly	65.	1919.22	{1866., 1966.}	2852.65	1.47013	Reff	0.65732	0.207656	{0.207071, 0.208243}	68.4086	ar	54 217.	101 453.	{101 337., 101 623.}	87.1237
3	R0	{1.68388, 4.76028}	{3., 20.}	{{3., 20.}, {3., 20.}}	{78.16, 320.143}	inf	45.	6940.15	{6934.28, 6948.13}	15 322.6	2.18816	newly	41.	2123.25	{2075., 2180.}	5078.66	1.71414	Reff	0.534935	0.207971	{0.206585, 0.210013}	61.1221	ar	68 299.	102 318.	{101 981., 102 692.}	49.8087
4	R0	{1.66014, 6.95383}	{2.99831, 20.}	{{3., 20.}, {3., 20.}}	{80.6061, 187.611}	inf	535.	6265.72	{6204.28, 6390.79}	1071.16	1.06858	newly	463.	1915.64	{1855., 1975.}	313.745	0.616596	Reff	0.564215	0.221472	{0.219147, 0.222284}	60.7469	ar	66 820.	98 982.2	{98 730., 99 161.}	48.1326
5	R0	{1.69984, 11.2038}	{2.30574, 20.}	{{2.26245, 20.}, {2.33723, 20.}}	{35.6447, 78.5109}	inf	2209.	6721.47	{6555.59, 6919.15}	204.277	0.483171	newly	1193.	2125.54	{2057., 2200.}	78.1676	0.250656	Reff	0.580631	0.425073	{0.412565, 0.436681}	26.7912	ar	68 462.	97 552.2	{96 920., 98 394.}	42.4911
6	R0	{1.65876, 6.66217}	{2.86307, 20.}	{{2.82481, 20.}, {2.87718, 20.}}	{72.603, 200.202}	inf	667.	7552.36	{7496.07, 7641.13}	1032.29	1.05394	newly	562.	2327.98	{2270., 2379.}	314.231	0.617158	Reff	0.560711	0.267258	{0.263875, 0.273149}	52.3359	ar	67 105.	98 651.7	{97 760., 98 960.}	47.011
7	R0	{1.69966, 5.89996}	{3., 20.}	{{3., 20.}, {3., 20.}}	{76.5059, 238.985}	inf	268.	7061.49	{7035.23, 7092.81}	2534.88	1.42076	newly	239.	2158.59	{2111., 2206.}	803.176	0.955704	Reff	0.52426	0.216678	{0.215897, 0.217715}	58.6698	ar	69 396.	99 766.1	{99 637., 99 917.}	43.7635
8	R0	{1.53428, 6.3734}	{2.19565, 17.6317}	{{2.19082, 17.5791}, {2.20011, 17.6889}}	{43.1063, 176.646}	inf	613.	6836.93	{6771.97, 6894.7}	1015.32	1.04739	newly	560.	2420.78	{2367., 2477.}	332.282	0.635702	Reff	0.635852	0.421646	{0.419841, 0.423842}	33.688	ar	59 855.	90 860.8	{90 672., 91 021.}	51.8016
9	R0	{1.58939, 3.48608}	{3., 20.}	{{3., 20.}, {3., 20.}}	{88.7517, 473.71}	inf	3.	10 441.5	{10 433.4, 10 448.4}	347 949.	3.54164	newly	4.	3209.04	{3143., 3272.}	80 126.	2.90427	Reff	0.594845	0.254004	{0.253624, 0.254443}	57.2991	ar	62 575.	97 854.6	{97 738., 97 979.}	56.3797
10	R0	{1.58528, 5.5593}	{1.97016, 20.}	{{1.96385, 20.}, {1.97427, 20.}}	{24.2782, 259.758}	inf	262.	11 400.7	{11 383.9, 11 415.7}	4251.4	1.63863	newly	240.	3751.25	{3681., 3810.}	1463.02	1.19393	Reff	0.591785	0.648444	{0.645222, 0.652739}	9.57422	ar	63 039.	84 444.6	{84 177., 84 718.}	33.9562
11	R0	{1.58305, 4.62721}	{3., 20.}	{{3., 20.}, {3., 20.}}	{89.5076, 332.226}	inf	119.	6743.64	{6728.47, 6761.11}	5566.93	1.75335	newly	130.	2055.93	{2001., 2117.}	1481.48	1.19897	Reff	0.585079	0.205371	{0.204117, 0.206216}	64.8986	ar	63 222.	101 971.	{101 788., 102 187.}	61.291
12	R0	{1.70234, 9.34626}	{2.00383, 20.}	{{1.99797, 20.}, {2.0085, 20.}}	{17.7104, 113.989}	inf	1435.	9904.4	{9885.21, 9922.25}	590.202	0.838975	newly	887.	3216.91	{3155., 3287.}	262.673	0.559466	Reff	0.572735	0.575944	{0.573092, 0.579739}	0.568748	ar	68 213.	86 065.6	{85 852., 86 268.}	26.1719
13	R0	{1.68556, 9.74954}	{2.32009, 20.}	{{2.2795, 20.}, {2.34981, 20.}}	{37.6451, 105.138}	inf	1573.	7136.07	{7044.02, 7303.61}	353.66	0.656693	newly	954.	2260.1	{2189., 2326.}	136.908	0.374457	Reff	0.561814	0.436396	{0.424852, 0.450854}	22.3237	ar	68 426.	98 085.8	{97 288., 98 943.}	43.3458
14	R0	{1.59876, 9.78897}	{2.22562, 19.8635}	{{2.20525, 20.}, {2.26484, 20.}}	{39.2089, 102.917}	inf	2224.	8479.81	{8369.35, 8652.69}	281.286	0.580731	newly	1422.	2742.46	{2671., 2818.}	92.8594	0.285143	Reff	0.640335	0.510026	{0.491865, 0.520906}	20.3502	ar	63 553.	95 943.4	{95 422., 97 064.}	50.966
15	R0	{1.43577, 5.12819}	{2.24437, 20.}	{{2.23993, 20.}, {2.24859, 20.}}	{56.3185, 290.001}	inf	365.	9969.93	{9941.23, 10 003.2}	2631.49	1.4364	newly	410.	3211.66	{3153., 3280.}	683.332	0.893893	Reff	0.682565	0.519586	{0.516747, 0.522252}	23.8774	ar	53 259.	93 233.6	{92 875., 93 624.}	75.0571
16	R0	{1.58866, 8.73191}	{2.18388, 20.}	{{2.13657, 20.}, {2.20739, 20.}}	{37.4667, 129.045}	inf	1770.	9633.14	{9494.85, 9920.97}	444.245	0.735739	newly	1205.	3096.34	{3021., 3175.}	156.958	0.409793	Reff	0.609442	0.508546	{0.483877, 0.519624}	16.5555	ar	64 349.	94 143.3	{93 289., 94 978.}	46.3011
17	R0	{1.43012, 3.8163}	{3., 20.}	{{3., 20.}, {3., 20.}}	{109.773, 424.068}	inf	129.	7978.43	{7958.07, 8002.76}	6084.83	1.79133	newly	198.	2444.96	{2383., 2494.}	1134.83	1.09154	Reff	0.671055	0.234905	{0.234099, 0.235744}	64.9947	ar	53 353.	100 872.	{100 707., 101 056.}	89.0646
18	R0	{1.47965, 5.16472}	{2.07345, 14.1333}	{{2.05819, 14.0865}, {2.10024, 14.2969}}	{40.1309, 173.651}	inf	307.	5018.72	{4976.53, 5085.34}	1534.76	1.21344	newly	352.	2167.6	{2123., 2215.}	515.795	0.789369	Reff	0.658962	0.444025	{0.432776, 0.447677}	32.6175	ar	56 086.	97 015.6	{96 586., 97 896.}	72.9766
19	R0	{1.57191, 5.2314}	{2.92105, 20.}	{{2.91384, 20.}, {2.92658, 20.}}	{85.8283, 282.307}	inf	180.	5868.29	{5853.79, 5902.26}	3160.16	1.51324	newly	174.	1801.29	{1756., 1850.}	935.224	1.01495	Reff	0.584248	0.235551	{0.234387, 0.237321}	59.683	ar	63 119.	100 767.	{100 540., 100 946.}	59.6468
20	R0	{1.54435, 5.32911}	{2.32053, 20.}	{{2.31534, 20.}, {2.32357, 20.}}	{50.2596, 275.297}	inf	264.	9711.45	{9696.71, 9730.85}	3578.58	1.56568	newly	266.	3072.4	{3019., 3134.}	1055.04	1.06255	Reff	0.594389	0.418872	{0.417003, 0.421991}	29.529	ar	61 870.	94 445.5	{94 245., 94 675.}	52.6516
21	R0	{1.57932, 4.90031}	{3., 20.}	{{3., 20.}, {3., 20.}}	{89.9552, 308.137}	inf	136.	7144.48	{7122.48, 7166.23}	5153.3	1.72043	newly	157.	2181.43	{2128., 2227.}	1289.45	1.14278	Reff	0.576847	0.219858	{0.21859, 0.221049}	61.8862	ar	63 595.	99 807.6	{99 423., 100 181.}	56.9425
22	R0	{1.47044, 4.07694}	{2.1278, 16.6812}	{{2.12417, 16.6336}, {2.1326, 16.7256}}	{44.7048, 309.16}	inf	134.	9295.24	{9237.78, 9352.5}	6836.75	1.84115	newly	178.	3509.97	{3440., 3578.}	1871.89	1.29483	Reff	0.652831	0.498096	{0.496179, 0.499748}	23.7023	ar	55 895.	88 195.	{88 030., 88 391.}	57.787
23	R0	{1.64997, 3.34546}	{3., 20.}	{{3., 20.}, {3., 20.}}	{81.8215, 497.825}	inf	4.	8262.11	{8259.1, 8265.24}	206 453.	3.31503	newly	5.	2530.78	{2471., 2586.}	50 515.6	2.70422	Reff	0.554456	0.219523	{0.219278, 0.219741}	60.4075	ar	66 404.	104 012.	{103 938., 104 075.}	56.6344
24	R0	{1.67405, 4.50254}	{3., 20.}	{{3., 20.}, {3., 20.}}	{79.2061, 344.194}	inf	61.	8656.86	{8647.12, 8669.69}	14 091.6	2.15203	newly	72.	2652.36	{2596., 2705.}	3583.83	1.56625	Reff	0.536115	0.226246	{0.224807, 0.227494}	57.799	ar	68 017.	100 811.	{100 433., 101 170.}	48.2144
25	R0	{1.50663, 3.1286}	{3., 20.}	{{3., 20.}, {3., 20.}}	{99.1199, 539.264}	inf	23.	8814.88	{8805.6, 8824.24}	38 225.6	2.58349	newly	31.	2708.24	{2648., 2754.}	8636.26	1.94127	Reff	0.620536	0.238144	{0.234626, 0.239024}	61.6228	ar	58 823.	101 211.	{101 001., 102 159.}	72.0596
26	R0	{1.69822, 6.34992}	{2.17795, 17.6522}	{{2.17691, 17.637}, {2.17876, 17.6718}}	{28.249, 177.991}	inf	344.	8778.87	{8752.92, 8810.59}	2452.	1.40688	newly	289.	3135.18	{3088., 3187.}	984.837	1.03533	Reff	0.518704	0.465382	{0.464511, 0.466546}	10.28	ar	69 816.	89 153.8	{88 991., 89 303.}	27.6982
27	R0	{1.55869, 4.92988}	{2.81559, 20.}	{{2.81355, 20.}, {2.8174, 20.}}	{80.6384, 305.689}	inf	156.	7684.49	{7672.55, 7699.48}	4825.96	1.69249	newly	170.	2370.14	{2318., 2414.}	1294.2	1.14427	Reff	0.591429	0.261626	{0.2608, 0.262498}	55.7638	ar	62 344.	99 132.2	{98 892., 99 399.}	59.0085
28	R0	{1.46085, 6.06899}	{1.92291, 13.3973}	{{1.91346, 13.2506}, {1.92977, 13.5587}}	{31.6294, 120.75}	inf	471.	4064.96	{3973.46, 4213.14}	763.049	0.935939	newly	430.	1858.78	{1798., 1928.}	332.274	0.635625	Reff	0.667083	0.483093	{0.477383, 0.488872}	27.5812	ar	55 484.	92 649.4	{92 087., 93 198.}	66.9839
29	R0	{1.63017, 6.25164}	{2.99307, 20.}	{{2.97106, 20.}, {3., 20.}}	{83.605, 219.916}	inf	417.	5993.68	{5960.14, 6044.17}	1337.33	1.15755	newly	364.	1835.96	{1777., 1890.}	404.385	0.702655	Reff	0.557469	0.226388	{0.219662, 0.231379}	59.3901	ar	66 231.	100 735.	{99 837., 101 191.}	52.0968
30	R0	{1.45173, 5.68165}	{2.1861, 20.}	{{2.17573, 20.}, {2.19334, 20.}}	{50.5858, 252.01}	inf	490.	9439.03	{9410.87, 9464.55}	1826.33	1.28473	newly	473.	3046.06	{2994., 3107.}	543.987	0.808838	Reff	0.663223	0.531877	{0.52747, 0.536969}	19.8042	ar	55 280.	90 456.7	{90 101., 90 814.}	63.6338
31	R0	{1.55808, 2.77316}	{3., 20.}	{{3., 20.}, {3., 20.}}	{92.5447, 621.199}	inf	8.	8697.98	{8695.19, 8700.98}	108 625.	3.03633	newly	17.	2668.6	{2612., 2731.}	15 597.6	2.19578	Reff	0.608757	0.228637	{0.228501, 0.228843}	62.4421	ar	60 940.	100 756.	{100 564., 100 925.}	65.3371
32	R0	{1.56797, 3.90327}	{3., 20.}	{{3., 20.}, {3., 20.}}	{91.3302, 412.391}	inf	58.	8248.38	{8239.09, 8258.34}	14 121.3	2.15294	newly	67.	2528.89	{2479., 2585.}	3674.46	1.57679	Reff	0.594323	0.227138	{0.226481, 0.227909}	61.7821	ar	62 228.	99 790.3	{99 585., 99 974.}	60.3624
33	R0	{1.70027, 5.43261}	{3., 20.}	{{3., 20.}, {3., 20.}}	{76.4426, 268.147}	inf	114.	6439.34	{6430.37, 6447.69}	5548.55	1.75194	newly	106.	1964.71	{1920., 2013.}	1753.5	1.26791	Reff	0.533137	0.201979	{0.201002, 0.202772}	62.115	ar	68 798.	104 354.	{104 013., 104 702.}	51.6811
34	R0	{1.61668, 6.86192}	{1.93724, 20.}	{{1.93243, 20.}, {1.94365, 20.}}	{19.8284, 191.464}	inf	584.	11 180.	{11 154.7, 11 228.5}	1814.38	1.28203	newly	504.	3690.41	{3622., 3753.}	632.224	0.864604	Reff	0.585885	0.671058	{0.665834, 0.674655}	14.5375	ar	64 693.	83 339.7	{83 022., 83 677.}	28.8234
35	R0	{1.55955, 3.45071}	{3., 20.}	{{3., 20.}, {3., 20.}}	{92.3632, 479.591}	inf	18.	9234.01	{9226.44, 9240.9}	51 200.1	2.71012	newly	21.	2829.61	{2774., 2885.}	13 374.3	2.12945	Reff	0.596107	0.230723	{0.229855, 0.231208}	61.295	ar	61 783.	103 265.	{102 997., 103 547.}	67.141
36	R0	{1.65068, 4.91307}	{2.99981, 20.}	{{3., 20.}, {3., 20.}}	{81.7319, 307.077}	inf	177.	8505.54	{8494.18, 8520.77}	4705.39	1.68173	newly	195.	2605.51	{2551., 2649.}	1236.16	1.1258	Reff	0.557286	0.234124	{0.23366, 0.23467}	57.9885	ar	66 453.	99 621.7	{99 493., 99 726.}	49.9131
37	R0	{1.46131, 3.06714}	{3., 20.}	{{3., 20.}, {3., 20.}}	{105.295, 552.073}	inf	14.	7689.62	{7682.62, 7697.}	54 825.8	2.73978	newly	26.	2354.52	{2308., 2413.}	8955.85	1.95686	Reff	0.636678	0.218143	{0.217795, 0.218425}	65.7373	ar	56 452.	101 979.	{101 752., 102 149.}	80.6481
38	R0	{1.71034, 5.2546}	{3., 20.}	{{3., 20.}, {3., 20.}}	{75.4037, 280.619}	inf	137.	6738.45	{6728.89, 6748.82}	4818.58	1.69184	newly	136.	2052.57	{2002., 2096.}	1409.24	1.17868	Reff	0.519003	0.204373	{0.202354, 0.206518}	60.6219	ar	69 816.	101 602.	{100 876., 102 201.}	45.5285
39	R0	{1.43565, 5.57989}	{2.01759, 13.5931}	{{2.00736, 13.5373}, {2.02215, 13.7989}}	{40.5351, 143.609}	inf	344.	4057.41	{4022.59, 4166.15}	1079.48	1.07165	newly	352.	1825.71	{1767., 1875.}	418.668	0.714781	Reff	0.663615	0.471603	{0.46894, 0.474447}	28.9342	ar	54 407.	91 726.6	{91 115., 91 978.}	68.5935
40	R0	{1.40327, 4.00764}	{2.42514, 15.8653}	{{2.41723, 15.7745}, {2.43955, 15.9407}}	{72.8203, 295.875}	inf	152.	5736.06	{5694.09, 5771.51}	3673.73	1.57676	newly	210.	2207.37	{2161., 2259.}	951.129	1.02159	Reff	0.680319	0.365067	{0.359697, 0.368216}	46.3389	ar	51 976.	101 452.	{101 165., 101 841.}	95.1898
41	R0	{1.63612, 4.26741}	{3., 20.}	{{3., 20.}, {3., 20.}}	{83.3606, 368.668}	inf	56.	7321.57	{7316.36, 7326.07}	12 974.2	2.11642	newly	65.	2237.39	{2189., 2285.}	3342.14	1.53676	Reff	0.573476	0.209507	{0.209036, 0.210019}	63.4673	ar	65 029.	103 442.	{103 162., 103 643.}	59.0704
42	R0	{1.57426, 6.50271}	{2.27998, 20.}	{{2.27412, 20.}, {2.28253, 20.}}	{44.8288, 207.564}	inf	569.	9561.94	{9540.93, 9586.47}	1580.48	1.22543	newly	480.	3060.77	{3008., 3116.}	537.66	0.804546	Reff	0.602202	0.487564	{0.485883, 0.490037}	19.0364	ar	62 522.	94 922.	{94 524., 95 290.}	51.8218
43	R0	{1.37818, 5.37636}	{2.34162, 20.}	{{2.33603, 20.}, {2.34603, 20.}}	{69.9069, 271.999}	inf	520.	8670.84	{8636.32, 8712.58}	1567.47	1.22205	newly	563.	2760.6	{2698., 2808.}	390.337	0.690442	Reff	0.701742	0.46231	{0.458762, 0.465137}	34.1197	ar	50 098.	99 736.9	{99 443., 100 038.}	99.0835
44	R0	{1.66606, 4.92641}	{3., 20.}	{{3., 20.}, {3., 20.}}	{80.0655, 305.975}	inf	140.	8424.64	{8412.77, 8438.6}	5917.6	1.77942	newly	138.	2576.27	{2516., 2627.}	1766.86	1.27106	Reff	0.551433	0.226921	{0.225995, 0.227926}	58.8488	ar	67 066.	100 071.	{99 899., 100 264.}	49.2128
45	R0	{1.623, 5.9449}	{2.91299, 20.}	{{2.8928, 20.}, {2.92124, 20.}}	{79.482, 236.423}	inf	438.	7550.13	{7514.13, 7634.18}	1623.77	1.23647	newly	424.	2317.84	{2257., 2367.}	446.66	0.737648	Reff	0.585189	0.251225	{0.249561, 0.254792}	57.0694	ar	64 554.	100 030.	{99 862., 100 203.}	54.9553
46	R0	{1.65235, 5.51234}	{2.14384, 16.978}	{{2.14121, 16.8142}, {2.14662, 17.0316}}	{29.7446, 208.}	inf	274.	9628.97	{9500.78, 9691.69}	3414.22	1.54582	newly	254.	3584.8	{3510., 3649.}	1311.34	1.14959	Reff	0.558213	0.50222	{0.500346, 0.503703}	10.0307	ar	66 509.	87 485.9	{87 257., 87 679.}	31.5399
47	R0	{1.62138, 8.82208}	{2.13757, 18.0355}	{{2.11974, 17.8227}, {2.17753, 18.4567}}	{31.8365, 104.436}	inf	1228.	6005.33	{5823.17, 6439.7}	389.033	0.689027	newly	834.	2106.09	{2024., 2215.}	152.529	0.402045	Reff	0.585561	0.466798	{0.460737, 0.474252}	20.2819	ar	65 598.	92 955.8	{92 416., 94 007.}	41.7052
48	R0	{1.64073, 6.69261}	{2.67139, 18.2752}	{{2.60217, 17.8939}, {3., 20.}}	{62.8171, 173.066}	inf	581.	6574.72	{6394.71, 7183.24}	1031.62	1.05337	newly	486.	2227.02	{2163., 2286.}	358.235	0.661003	Reff	0.561064	0.35542	{0.279494, 0.37196}	36.6526	ar	66 438.	99 143.4	{98 449., 101 505.}	49.227
49	R0	{1.65556, 3.796}	{3., 20.}	{{3., 20.}, {3., 20.}}	{81.2076, 426.87}	inf	19.	9575.48	{9570.6, 9581.2}	50 297.3	2.70241	newly	19.	2926.97	{2869., 2983.}	15 305.1	2.18761	Reff	0.553586	0.229277	{0.228226, 0.230064}	58.5833	ar	66 575.	103 013.	{102 637., 103 347.}	54.7327
50	R0	{1.56828, 3.65713}	{3., 20.}	{{3., 20.}, {3., 20.}}	{91.2924, 446.877}	inf	15.	7489.4	{7484.88, 7496.04}	49 829.4	2.69836	newly	19.	2283.57	{2230., 2337.}	11 918.8	2.0798	Reff	0.584592	0.209954	{0.209574, 0.210429}	64.0854	ar	62 761.	103 893.	{103 670., 104 113.}	65.5374
